# Partial Purification and Characterization of Rhodanese from Rainbow Trout (*Oncorhynchus mykiss*) Liver

**DOI:** 10.1100/2012/648085

**Published:** 2012-05-02

**Authors:** Hossein Tayefi-Nasrabadi, Reza Rahmani

**Affiliations:** Department of Basic Sciences, College of Veterinary Medicine, University of Tabriz, P.O. Box 51666-16471, Tabriz, Iran

## Abstract

Cyanide is one of the most toxic substances present in a wide variety of food materials that are consumed by animals. Rhodanese, a ubiquitous enzyme, can catalyse the detoxification of cyanide by sulphuration reaction. In this study, rhodanese was partially purified and characterized from the liver tissue homogenate of the rainbow trout. The enzyme was active in a broad range of pH, from 5 to 12. The optimal activity was found at a high pH (pH 10.5), and the temperature optimum was 25°C. The enzyme was heat labile, losing > 50% of relative activity after only 5 min of incubation at 40°C. The *K*_m_ values for KCN and Na_2_S_2_O_3_ as substrates were 36.81 mM and 19.84 mM, respectively. Studies on the enzyme with a number of cations showed that the activity of the enzyme was not affected by Sn^2+^, but Hg^2+^, Ba^2+^, Pb^2+^, and Ca^2+^ inhibited and Cu^2+^ activated the enzyme with a concentration-dependent manner.

## 1. Introduction

Rhodanese (thiosulphate: cyanide sulphurtransferase, EC 2.8.1.1) is a sulphur transferase that catalyses the formation of thiocyanate from free cyanide and a sulphur donor. It is a ubiquitous enzyme that is active in bacteria, yeast, plants, and animals [1, 2]. Beside cyanide detoxification, many other physiological functions have been proposed for rhodanese enzyme: it supplies sulphide for the formation of iron-sulphur centres, thiamine biosynthesis, maintains the sulphane pool, participates in selenium metabolism, and functions as a thioredoxin oxidase [3–5]. Intracellular study of the enzyme revealed that it is present in the cytosol, mitochondrion, and nucleus [6].

In aquatic organisms, freshwater fish are the most cyanide-sensitive group with high mortality at free cyanide concentrations above 20 *μ*g/L. Studies on cyanide toxicity in fish indicate that it has adverse effects on swimming and reproduction at >5 *μ*g/L [7].

The rainbow trout (*Oncorhynchus mykiss*) is a species of salmonid native to tributaries of the Pacific Ocean in Asia and North America. This species constitutes the major group of cultured freshwater fish with great commercial importance. This paper reports on the partial purification and characterization of rhodanese isolated in the liver of rainbow trout.

## 2. Materials and Methods

### 2.1. Chemicals

Ammonium sulphate (enzyme grade), potassium cyanide, and sodium thiosulphate (pentahydrate) were obtained from Sigma Chemical Company, St. Louis, USA. All other reagents were of analytical grade and were obtained from Merck (Darmstadt, Germany).

### 2.2. Enzyme Preparation

Live fish weighing 350–450 g were obtained from local fish dealers, transported live to the laboratory, sacrificed, and then dissected. Liver was quickly removed and washed with physiological saline. Liver extracts were prepared by freezing the samples in liquid nitrogen, homogenizing with a hand homogenizer, and suspending the homogenates in 25 mM sodium phosphate buffer, pH 7.2. The suspensions were centrifuged for 10 min at 4,000 rpm. Supernatant was brought to 65% ammonium sulphate saturation (430 g/L) by the addition of solid ammonium sulphate over a period of 1 hour with continuous stirring and then left overnight. The resulting precipitate was collected by centrifugation at 4,000 rpm for 15 min and immediately dialyzed against several changes of a 50 mM citrate buffer, pH 5.0. The dialysate was centrifuged at 13,400 rpm for 15 min to remove insoluble materials.

### 2.3. Enzyme Assay

Rhodanese was assayed by the modified method of Sorbo [8]. The reaction mixture consisted of a 25 mM citrate-phosphate-borate buffer, at given pH, 50 mM KCN, 50 mM Na_2_S_2_O_3_, and 50 *μ*L enzyme in a total volume of 1.75 mL. The mixture was incubated for 2 min at 25°C, and the reaction was stopped by the addition of 0.25 mL of 37% formaldehyde, followed by the addition of 1 mL of ferric nitrate solution containing 0.025 g Fe(NO3)3 9H2O in 0.74 mL water and 0.26 mL concentrated nitric acid. The absorbance was then read at 460 nm. Reaction velocity was computed from linear slopes of absorbance-time curve. One unit of rhodanese activity was defined as micromole of thiocyanate formed per min. Protein concentrations were determined by the method of Lowry et al. [9], using crystalline bovine serum albumin as standard.

### 2.4. Kinetic Studies

The *K*_*m*_ for cyanide ion was determined by varying the concentration of KCN between 10 mM and 50 mM at 50 mM Na_2_S_2_O_3_. Also, *K*_*m*_ for sodium thiosulphate was determined by varying the Na_2_S_2_O_3_ concentration from 10 mM to 50 mM at 50 mM of KCN.

### 2.5. Effect of pH

The effect of pH on the rainbow trout liver rhodanese was assessed by assaying the enzyme at different pH using TS buffer (citrate-phosphate-borate, 25 mM, pH 5–12). 

### 2.6. Effect of Temperature

For determination of optimum temperature of enzyme activity, 50 *μ*L of the enzyme was assayed at temperatures between 20° and 70°C. The reaction mixture was first incubated at the selected temperature for 10 min before initiating the reaction by the addition of the enzyme that had been equilibrated at the same temperature.

Thermal stability of liver rhodanese was studied by incubating aliquots of enzyme at various temperatures (25, 30, 40, 50, 60 and 70°C) up to 60 min in a thermostatic water bath and measuring their activity at room temperature after brief cooling in ice. The incubation was carried out in sealed vials to prevent change of volume of the sample and, hence, the enzyme concentration due to evaporation.

### 2.7. Effect of Cations

Rhodanese activity was measured in the presence (final concentration 0.5 or 1 mM) and absence of various ionic compounds (SnCl_2_, HgCl_2_, BaCl_2_, PbCl_2_, CaCl_2_, and CuCl_2_) under the standard conditions.

 All assays were done at least in 3 separate experiments, and the mean values of data were reported.

## 3. Results

**Figure 1:**
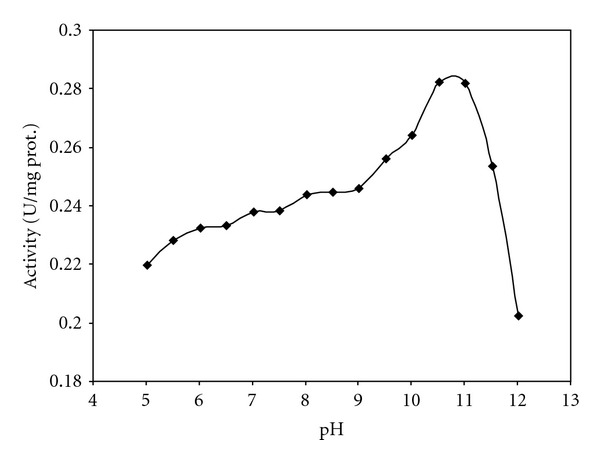
Effect of pH on rainbow trout liver rhodanese. The assay mixture contained 25 mM citrate-phosphate-borate buffer, at given pH, and 50 mM KCN, 50 mM Na_2_S_2_O_3_, and 50 *μ*L enzyme solution in a total volume of 1.75 mL.

**Figure 2:**
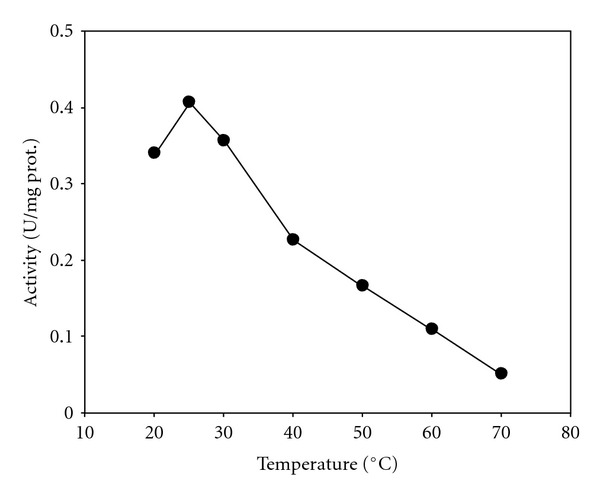
Effect of temperature on rainbow trout liver rhodanese.

**Figure 3:**
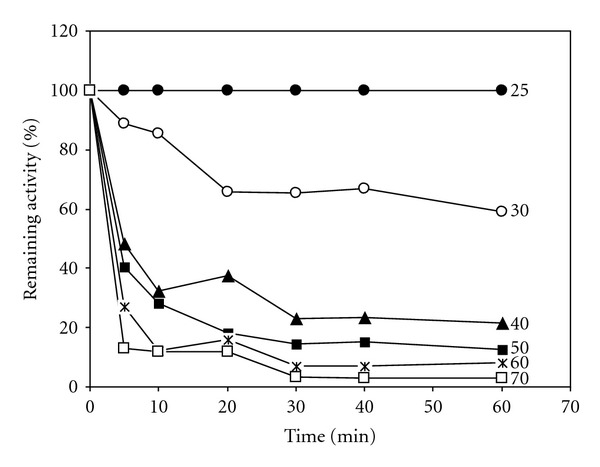
Effect of heat treatment on rainbow trout rhodanese activity as a function of treatment time at different temperatures. The remaining activity was expressed as a percentage of activity of the enzyme incubated at 25°C.

**Figure 4:**
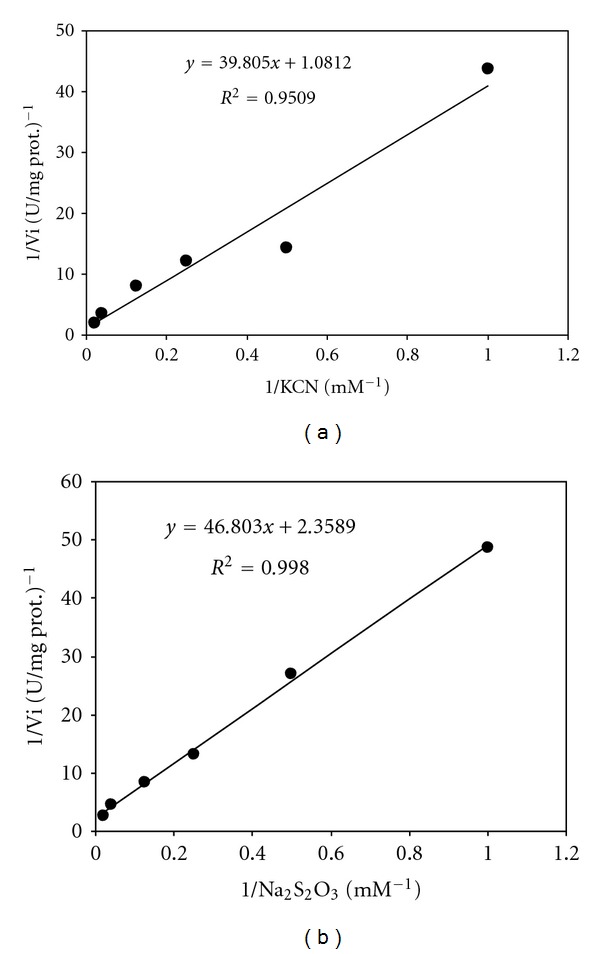
Lineweaver-Burk plot for the determination of the kinetic parameters of rainbow trout rhodanese. The enzyme was assayed at varying concentrations of Na_2_S_2_O_3_ at 50 mM KCN (a) and varying concentrations of KCN at 50 mM Na_2_S_2_O_3_ (b). The reaction mixture consisted of a 25 mM citrate-phosphate-borate buffer, pH 10.5, and 50 *μ*L enzyme in a total volume of 1.75 mL at 25°C.

Table 1 shows the results of the partial purification of rhodanese from the liver of rainbow trout. The specific activity of enzyme after partial purification using ammonium sulphate precipitation and dialysis was 0.206 U/mg. The effect of pH on the rate of rhodanese activity is shown in Figure 1. Rhodanese activity was found in pH ranging from 5 to 12. An optimum pH of 10.5 was obtained. The effect of temperatures between 20 and 70°C on the rhodanese activity showed that optimum temperature for the enzyme was 25°C (Figure 2). The enzyme was incubated at different temperatures for 60 min and, after brief cooling in ice, the residual enzyme activity was measured (Figure 3). It was found that the enzyme was stable at 25°C for 60 min but was unstable at temperatures ≥30°C. Michaelis constants (*K*_*m*_) and maximum reaction velocities (*V*_max⁡_) of the rainbow trout liver rhodanese were determined using Lineweaver-Burk plot under optimum conditions (Figures 4(a) and 4(b)). The *K*_*m*_ values for KCN and Na_2_S_2_O_3_ were 36.81 mM and 19.84 mM, and the *V*_max⁡_ values for these substrates (KCN and Na_2_S_2_O_3_) were 0.924 and 0.423 U/mg, respectively. A comparison of rainbow trout rhodanese with some other rhodanese preparations is shown in Table 2. The result of the effect of various cations on the activity of rhodanese is presented in Table 3.

## 4. Discussion

Cyanide is a potent cytotoxic agent that kills the cell by inhibiting cytochrome oxidase of the mitochondrial electron transport chain. When ingested, cyanide activates the body own mechanisms of detoxification, resulting in the transformation of cyanide into a less toxic compound, thiocyanate. Rhodanese and 3-mercaptopyruvate sulphurtransferase represent the chief enzymes of cyanide detoxification [1, 2]. Rainbow trout constitutes the major group of cultured freshwater fish with great commercial importance. This research shows the existence of rhodanese in the rainbow trout liver homogenate. The enzyme was partially purified by ammonium sulphate precipitation and dialysis. As shown in Figure 1, optimum pH for rainbow trout liver rhodanese was 10.5. This value is greater than the values found for rhodanese of bovine liver (between 8.0 and 9.0) [8, 14], mouse liver (9.4) [13], African catfish liver (6.5) [10], fruit bat liver (9) [12], and mudskipper liver (8) [11]. Chew and Boey [16], working on the tapioca leaf, obtained a high pH value of 10.2–11, which is very close to the pH optimum of rainbow trout liver rhodanese.

Different optimum temperatures have been reported for rhodanese from different organisms. Akinsiku et al. [10], reported 40°C for the rhodanese from the liver of the African catfish from Asejire Lake. Himwich and Saunders [17] also obtained an optimum temperature of between 38°C and 40°C for bovine liver rhodanese. Agboola and Okonji [12] reported 35°C for the rhodanese in the cytosolic fraction of fruit bat liver. In an earlier study, Emuebie et al. [11] obtained the high optimum temperature of 50°C for mudskipper liver rhodanese. The optimum temperature obtained was 25°C for rhodanese from the liver of the rainbow trout. The thermal stability experiment showed that the enzyme was heat labile, losing about 52% of relative activity after only 5 min of incubation at 40°C. The enzyme retained 59, 21, 12, 8, and 3% of its activity at 30, 40, 50, 60, and 70°C when heated for 1 hour. Ploegman et al. [18] reported that rhodanese consists of two equally sized, similarly folded domains stabilized by extensive hydrophobic interactions. The active site resides in a deep cavity formed by the juxtaposition of these domains. This enzyme appears sensitive to thermal inactivation, a process which apparently results from thermally induced transitions of the native structure, which lead to the exposure of hydrophobic surfaces and irreversible protein association [19].

The apparent *K*_*m*_ values, as determined by the Lineweaver-Burk plot for KCN and Na_2_S_2_O_3_, were 36.81 and 19.84 mM, respectively, for liver of the rainbow trout. These values are compared to those reported in earlier studies in Table 2. As shown in Table 2, the *K*_*m*_ values of rainbow trout liver rhodanese are higher than those of African catfish liver, mudskipper liver, fruit bat liver, mouse liver, bovine liver, and human liver, indicating that the affinity of the rainbow trout enzyme for these substrates is less than that of the other enzymes and that it would catalyze the detoxification reaction with less efficiency. As reported by Agboola and Okonji [12] this less effective enzymatic system may be due to a lower exposure to cyanide, in contrast to its mammalian animals, which are continually exposed through their diet. However, it should be noted that cyanide detoxification is a secondary benefit of rhodanese, which has a number of major physiological roles, including the supply of sulphide for the formation of iron-sulphur centres for the electron transport chain.

Specific binding sites for some metal ions have been crystallographically documented for a reduced number of proteins, including rhodanese [20]. In some of these cases, the binding of the cation has been found to promote functional effects [21]. In this study, the effect of some metal ions such as Sn^2+^, Hg^2+^, Ba^2+^, Pb^2+^, Cu^2+^, and Ca^2+^ on activity of rainbow trout liver rhodanese was also studied. Results show that between these cations, only Cu^2+^ could activate the enzyme, but Hg^2+^, Ba^2+^, Pb^2+^, Cu^2+^, and Ca^2+^ inhibited the enzyme by dose-dependent manner, respectively.

A great deal of the beneficiary as well as detrimental effects of transition metals comes from these metals ability to coordinate to protein residues and any protein may have more than one binding site for a given metal. Metal ion is known to bind to peptides and proteins, and a number of potential metal-binding residues, including cysteine, histidine, tyrosine, and asparagines, have been identified. Changes in the tertiary and secondary structure of the enzyme were also reported after incubation of protein with metal ions [22]. The inhibition or activation of rainbow trout liver rhodanese by these cations is probably due to the interaction of these metal ions with sulphydryl groups at the enzyme catalytic site [23] or induction of changes in conformation of enzyme. Further experimental studies are required to elucidate the mechanism of activation or inhibition of the enzyme with these metal ions. In conclusion, the biochemical parameters obtained would confirm the enzyme to function in the conversion of cyanide, from various sources in the water, to thiocyanate and thereby presumably in the detoxification of the poisonous cyanide, ultimately improving the survival of the fish in polluted water.

## Figures and Tables

**Table 1:** Summary of the partial purification of rainbow trout liver rhodanese.

Fraction	Volume (mL)	Total activity (U·mL^−1^)	Total protein (mg)	Specific activity (U·mg^−1^ pro.)	Yield (%)	Purification fold
Crude extract	105	157.99	1295.76	0.121	100	1
65% ammonium sulphate precipitation	45	49.83	241.83	0.206	31.5	1.7

**Table 2:** Comparison of *K*_*m*_ values for rainbow trout liver with other liver rhodanese preparations.

Substrate	*K*_*m*_ (mM)						
	Rainbow trout	African catfish^a^	Mudskipper^b^	Fruit bat^c^	Mouse^d^	Bovine^e^	Human^f^
KCN	36.81	25.4	33.3	13.36	12.5	19.0	9.5
Na_2_S_2_O_3_	19.84	18.6	14.29	19.15	8.3	6.7	4.5

Data obtained from references [10–15].

**Table 3:** Effect of cations on rainbow trout liver rhodanese activity.

Salt	Enzyme activity (%)	
	0.5 mM	1.0 mM
None	100	100
SnCl_2_	100	100
CuCl_2_	206	351
HgCl_2_	95	92
CaCl_2_	94	76
BaCl_2_	93	87
PbCl_2_	98	66
